# Vitamin D’s Role in Reducing Risk of SARS-CoV-2 and COVID-19 Incidence, Severity, and Death

**DOI:** 10.3390/nu14010183

**Published:** 2021-12-31

**Authors:** William B. Grant

**Affiliations:** Sunlight, Nutrition and Health Research Center, P.O. Box 641603, San Francisco, CA 94164-1603, USA; williamgrant08@comcast.net; Tel.: +1-415-409-1980

The article by D’Avolio and colleagues [1] on patients with a positive polymerase chain reaction (PCR) test for SARS-CoV-2 was the first to report that 25-hydroxyvitamin D [25(OH)D] concentrations were lower in PCR-positive patients than in PCR-negative patients or in historical controls. As a result, that report has the most citations in its category (201 according to SCOPUS on 17 December 2021). Thus, the article likely helped considerably stimulate investigation of vitamin D’s role in reducing risk of SARS-CoV-2 infection and ensuing COVID-19.

A later article on 25(OH)D concentration and risk of SARS-CoV-2 positivity was based on more than 190,000 PCR tests on patients in the U.S. with serum 25(OH)D concentration measurements from the previous 12 months on file by quest diagnostics [2]. According to that report, positivity was inversely correlated with seasonally adjusted 25(OH)D concentration, posing a risk for patients with concentrations of ~55 ng/mL have about half the positivity of those with concentrations of <20 ng/mL.

A recent meta-analysis involving 76 studies reported inverse correlations for COVID-19 risk with respect to serum 25(OH)D concentrations for risk of developing the disease, its severity, and risk of death [3], thus offering support for the role of vitamin D in reducing risk of COVID-19.

However, because of the concern that having an acute inflammatory illness could lower serum 25(OH)D concentrations [4], observational studies of COVID-19 using 25(OH)D concentrations measured at or near time of diagnosis have been questioned. The data in Figure 3 of reference [3] can be used to address that concern. The 19 studies can be divided into four categories: 25(OH)D at time of diagnosis (seven studies); within 1 year prior (eight); preceding 10 years (one); and 10–15 years prior (three). The mean weighted odds ratios for each time are 2.08, 1.76, 1.27, and 1.04, respectively. Those values are consistent with a longer interval between blood draw and health outcome, which are associated with worse health outcomes due to changes in 25(OH)D concentrations. That effect is most pronounced for diseases that can develop rapidly, such as breast cancer [5]. Although the present editorial does not rule out reduction in 25(OH)D due to COVID-19, the times before diagnosis are independent of COVID-19 and support the role of vitamin D in reducing risk.

The innate immune system mechanisms by which vitamin D reduces risk of SARS-CoV-2 infection and COVID-19 appear to include reduced viral viability and replication by inducing cathelicidin and defensins as well as reduced production of proinflammatory cytokines and the risk of the cytokine storm [6]. Additional mechanisms were suggested later [7]. The innate immune system is not sensitive to the variant of SARS-CoV-2 involved. That is important because the virus mutates easily, thereby reducing the adaptive immune system’s ability to respond effectively. Thus, vitamin D can serve as an extra measure of protection as vaccine effectiveness wanes. The recommended serum 25(OH)D concentration for prevention is 40 to ≥60 ng/mL, which could be achieved with 5000 to 10,000 IU/d vitamin D_3_, i.e., a safe dose range [8].

Thus, it seems likely that raising serum 25(OH)D concentrations after SARS-CoV-2 infection or development of COVID-19 could reduce the progression and severity of COVID-19. Two approaches were attempted with mixed results. The most comprehensive trial of vitamin D_3_ supplementation with COVID-19 patients was a trial conducted in Turkey [9]. Of 867 COVID-19 patients, 160 patients with initial 25(OH)D concentrations of <30 ng/mL were treated with 224,000 to 500,000 IU over periods from 3 to 7 days to achieve >30 ng/mL. The only clinically significant benefit in comparison with untreated patients with 25(OH)D <30 ng/mL was a reduction in hospital stays over 8 days. However, the length of hospital stay was not significantly different between the two groups. That trial probably failed to achieve significant beneficial effects because the body takes several days to convert vitamin D to 25(OH)D, during which time the disease progresses, and because the achieved 25(OH)D concentrations were only 32 ± 12 ng/mL on day 7 and 36 ± 11 ng/mL on day 14.

An alternate approach is to supplement with calcifediol [25(OH)D], which raises serum 25(OH)D concentrations in hours. A successful pilot randomized controlled trial of calcifediol treatment was reported from Barcelona in 2020 [10]. That trial included 76 consecutive COVID-19 patients with acute respiratory tract infection and a positive PCR test for SARS-CoV-2. In addition to standard hospital care, 50 patients were assigned to receive calcifediol treatment: 0.532 mg on the first day, 0.266 mg on days 3 and 7, and then weekly until discharge or ICU admission. Calcifediol is about 3.2 times more efficient at raising serum 25(OH)D concentrations than vitamin D_3_ [11]. Thus, 0.266 mg of calcifediol is equivalent to ~34,000 IU of vitamin D_3_. Only 1 of 50 patients treated with calcifediol required ICU treatment, as opposed to 13 of 26 not receiving calcifediol (2 of whom died).

An observational study in Spain involved 537 patients hospitalized with COVID-19 between 5 February and 5 May 2020, of whom 79 were treated with calcifediol [12]. The odds ratio for mortality within 30 days, adjusted for potential confounders, was 0.16 (95% confidence interval, 0.03 to 0.80).
